# Supplementary material for: AliSim: A Fast and Versatile Phylogenetic Sequence Simulator for the Genomic Era
Source: Mol Biol Evol. 2022 May 3;39(5):msac092. doi: 10.1093/molbev/msac092 (PMC9113491; doi:10.1093/molbev/msac092)
Supplement: msac092_Supplementary_Data [file msac092_supplementary_data.pdf]

SUPPLEMENTARY MATERIAL FOR

AliSim: a fast and versatile phylogenetic sequence simulator for the genomic era

Nhan Ly-Trong, Suha Naser-Khdour, Robert Lanfear, Bui Quang Minh

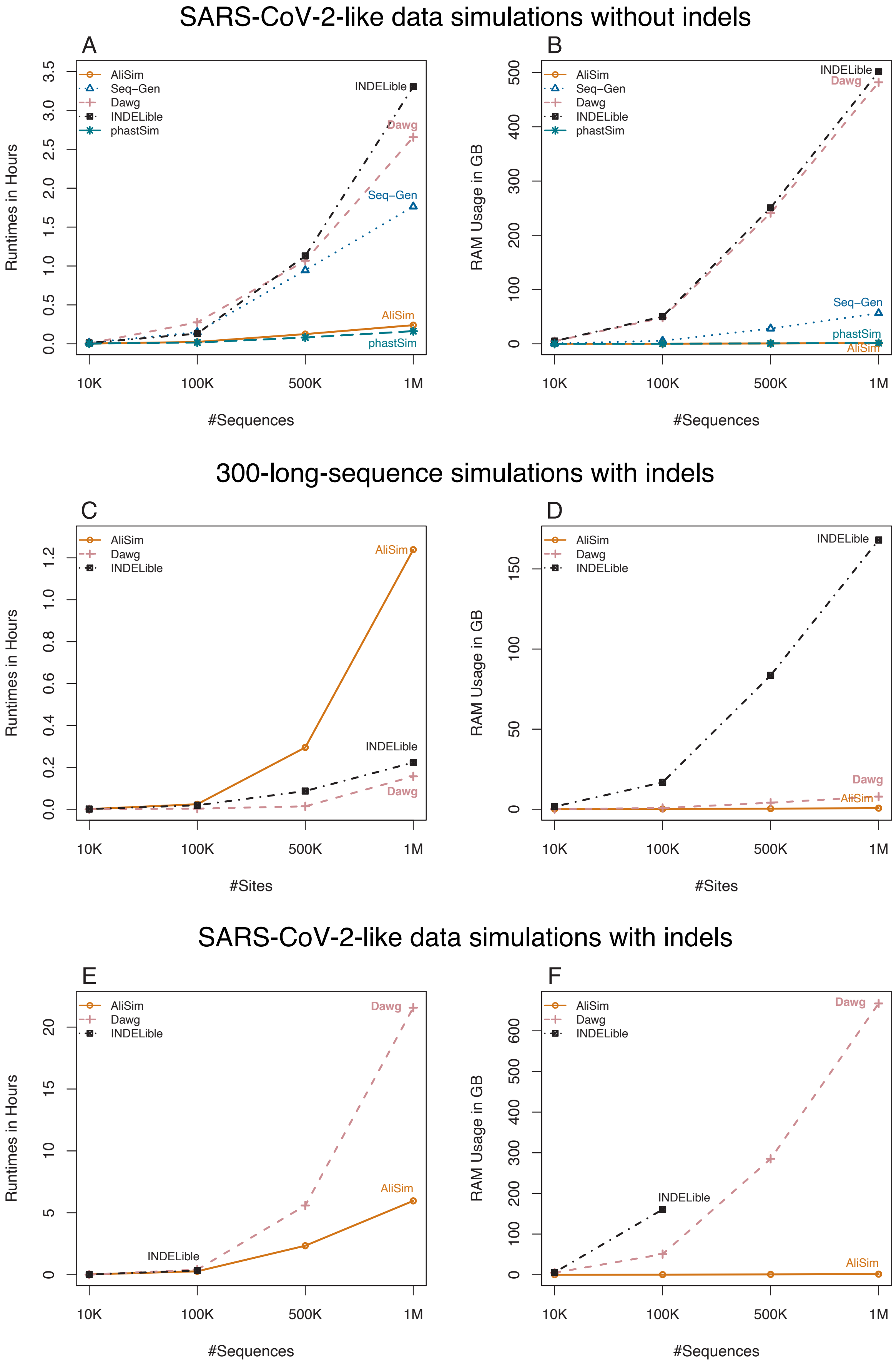
SARS-CoV-2-like data simulations without indels
A
B
AliSim
Seq−Gen
Dawg
INDELible
phastSim
Runtimes in Hours
RAM Usage in GB
#Sequences
10K
100K
500K
1M
300-long-sequence simulations with indels
C
D
#Sites
SARS-CoV-2-like data simulations with indels
E
F

Figure S1. Runtimes and peak memory consumptions of five software AliSim, Seq-Gen, Dawg, INDELible, and phastSim for SARS-CoV-2-like data simulations without indels (sub-panels A and B), for 300-long-sequence (300 sequences and varying the root sequence length between 10K and 1M sites; sub-panels C and D) simulations with indels, and for SARS-CoV-2-like data simulations with indels (sub-panels E and F). The SARS-CoV-2-like trees were drawn under a Yule process with the birth rate equal to one divided by the output sequence length so that there was, on average, only one mutation per branch (De Maio et al. 2021).

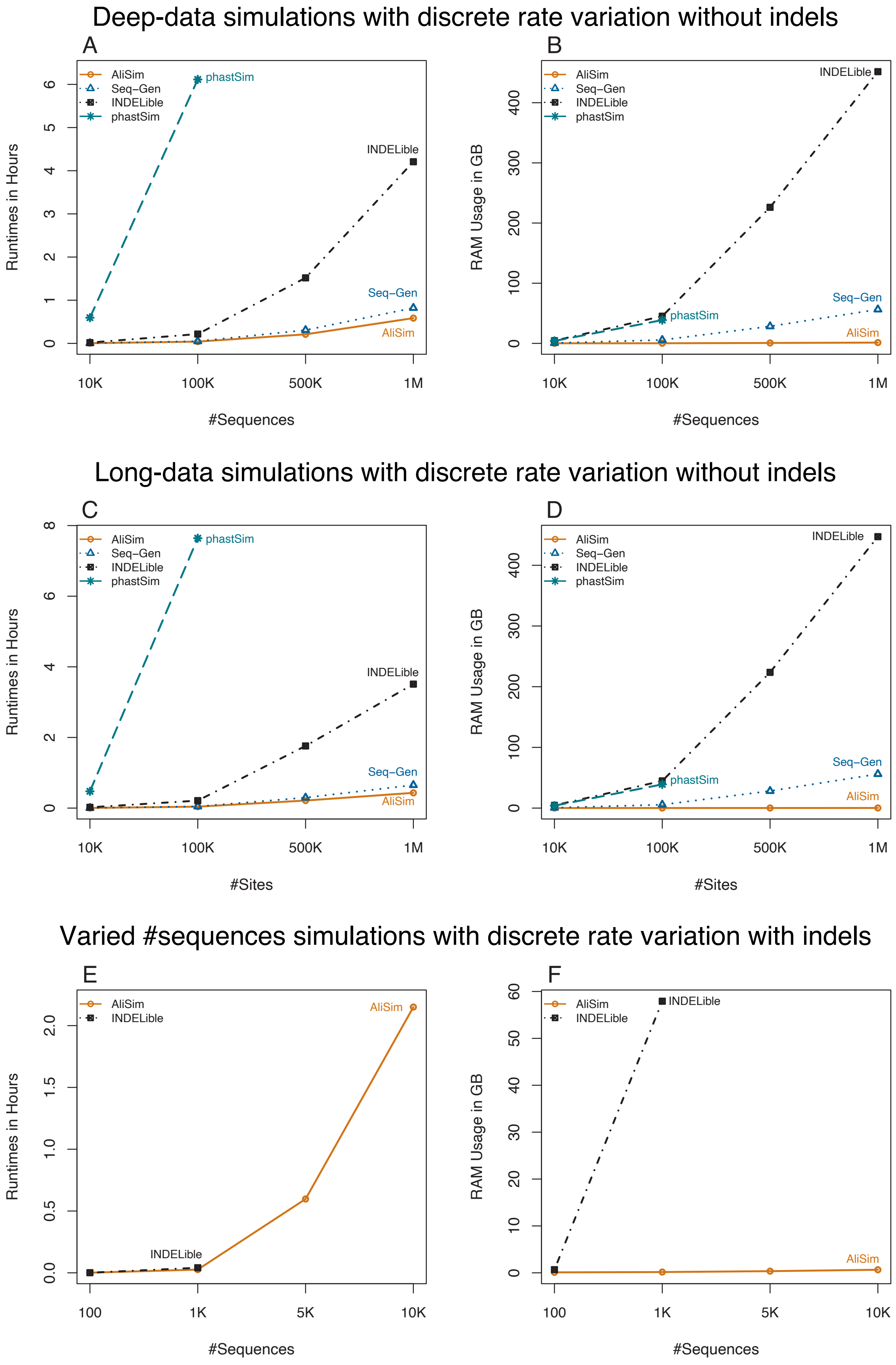
Deep-data simulations with discrete rate variation without indels
A
Runtimes in Hours
AliSim
Seq−Gen
INDELible
phastSim
#Sequences
10K
100K
500K
1M
B
RAM Usage in GB
#Sequences
Long-data simulations with discrete rate variation without indels
C
Runtimes in Hours
#Sites
D
RAM Usage in GB
#Sites
Varied #sequences simulations with discrete rate variation with indels
E
Runtimes in Hours
#Sequences
100
1K
5K
10K
F
RAM Usage in GB
#Sequences

Figure S2. Runtimes and peak memory consumptions of four software AliSim, Seq-Gen, INDELible, and phastSim in tests with discrete rate variation for deep-data simulations without indels (sub-panels A and B), long-data simulations without indels (sub-panels C and D), and varied #sequences simulations with indels (sub-panels E and F). For the model of sequence evolution, we applied the GTR model with a 0.2 proportion of invariant sites and four discrete rates across sites from a Gamma model (shape parameter of 0.5). We excluded Dawg because it does not support discrete rate variation. For INDELible, we used Method 1, which is more efficient than Method 2 in simulations with discrete rate variation (Fletcher and Yang 2009). For phastSim, we manually generated four discrete rates from a Gamma distribution before running the simulations.

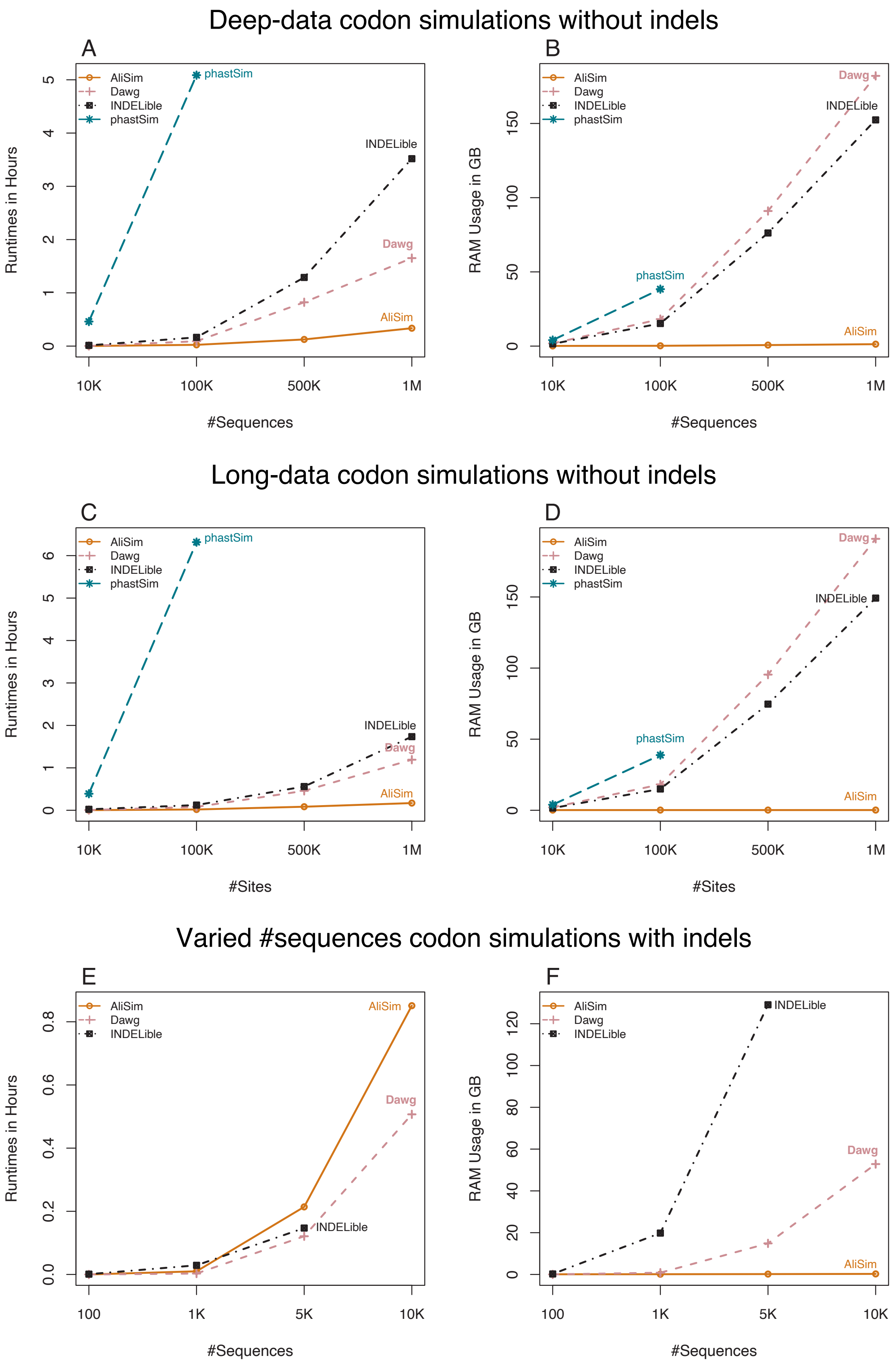
Deep-data codon simulations without indels
A
B
AliSim
Dawg
INDELible
phastSim
Runtimes in Hours
RAM Usage in GB
#Sequences
10K
100K
500K
1M
Long-data codon simulations without indels
C
D
#Sites
Varied #sequences codon simulations with indels
E
F
100
1K
5K
10K

Figure S3. Runtimes and peak memory consumptions of four software AliSim, Dawg, INDELible, and phastSim for deep-data codon simulations without indels (sub-panels A and B), long-data codon simulations without indels (sub-panels C and D), and varied #sequences simulations with indels (sub-panels E and F). For the model of sequence evolution, we applied the GY model (Goldman and Yang 1994). We excluded Seq-Gen because it does not support codon models.

Table S1. Validation results of AliSim on 287 simulations across a wide range of substitution models and insertion-deletion rates of 0.0, 0.02, 0.04, 0.06, 0.08 and 0.1.

No	Data Type	Indel-rate	The true (input) model	The best-fit model	Rank of the true model	RF distance	Inferred tree length	True tree length
**Substitution models**								
1	DNA	0/0	GTR{2.0,36.5,50.3,10.0,20.7}+F{0.3,0.1,0.2,0.4}	GTR+F+ASC	2	4	21.278	21.385
2	DNA	0/0	GTR{2.0,36.5,50.3,10.0,20.7}+F{0.2,0.1,0.3,0.4}+I{0.2}	GTR+F+I	1	0	21.101	19.890
3	DNA	0/0	GTR{2.0,36.5,50.3,10.0,20.7}+F{0.4,0.1,0.2,0.3}+G{0.5}	GTR+F+G4	1	0	19.654	19.769
4	DNA	0/0	GTR{2.0,36.5,50.3,10.0,20.7}+F{0.1,0.2,0.4,0.3}+I{0.2}+G{0.5}	GTR+F+I+G4	1	2	20.309	20.194
5	DNA	0/0	JC	JC	1	4	19.733	19.706
6	DNA	0/0	JC+I{0.2}	JC+I	1	2	19.695	21.315
7	DNA	0/0	JC+G{0.5}	JC+G4	1	6	21.483	21.543
8	DNA	0/0	JC+I{0.2}+G{0.5}	JC+I+G4	1	0	18.669	18.706
9	DNA	0/0	F81+F{0.3,0.1,0.2,0.4}	F81+F+ASC	2	4	20.174	20.150
10	DNA	0/0	F81+F{0.2,0.1,0.3,0.4}+I{0.2}	F81+F+I	1	2	19.627	19.341
11	DNA	0/0	F81+F{0.4,0.1,0.2,0.3}+G{0.5}	F81+F+G4	1	0	19.646	19.811
12	DNA	0/0	F81+F{0.1,0.2,0.4,0.3}+I{0.2}+G{0.5}	F81+F+I+G4	1	2	18.882	18.877

13	DNA	0/0	K80{2.0}	K2P	1	0	21.212	21.255
14	DNA	0/0	K80{2.0}+I{0.2}	K2P+I	1	2	16.908	17.401
15	DNA	0/0	K80{2.0}+G{0.5}	K2P+G4	1	0	19.947	19.986
16	DNA	0/0	K80{2.0}+I{0.2}+G{0.5}	K2P+I+G4	1	6	20.690	20.546
17	DNA	0/0	HKY{2.0}+F{0.3,0.1,0.2,0.4}	HKY+F+ASC	2	2	22.092	22.037
18	DNA	0/0	HKY{2.0}+F{0.2,0.1,0.3,0.4}+I{0.2}	HKY+F+I	1	2	19.521	21.586
19	DNA	0/0	HKY{2.0}+F{0.4,0.1,0.2,0.3}+G{0.5}	HKY+F+G4	1	0	17.866	18.032
20	DNA	0/0	HKY{2.0}+F{0.1,0.2,0.4,0.3}+I{0.2}+G{0.5}	TPM2u+F+I+G4	2	2	20.959	21.021
21	DNA	0/0	TN{2.0,4.0}+F{0.3,0.1,0.2,0.4}	TN+F+ASC	2	0	20.641	20.676
22	DNA	0/0	TN{2.0,4.0}+F{0.2,0.1,0.3,0.4}+I{0.2}	TN+F+I	1	2	20.969	20.645
23	DNA	0/0	TN{2.0,4.0}+F{0.4,0.1,0.2,0.3}+G{0.5}	TN+F+G4	1	4	19.989	20.025
24	DNA	0/0	TN{2.0,4.0}+F{0.1,0.2,0.4,0.3}+I{0.2}+G{0.5}	TN+F+I+G4	1	4	21.945	21.939
25	DNA	0/0	TNe{2.0,4.0}	TNe+ASC	2	0	20.485	20.511
26	DNA	0/0	TNe{2.0,4.0}+I{0.2}	TNe+I	1	4	15.721	19.082
27	DNA	0/0	TNe{2.0,4.0}+G{0.5}	TNe+G4	1	0	19.682	18.843
28	DNA	0/0	TNe{2.0,4.0}+I{0.2}+G{0.5}	TNe+I+G4	1	4	21.828	21.728
29	DNA	0/0	K81{2.0,4.0}	K3P+ASC	2	0	20.321	20.325
30	DNA	0/0	K81{2.0,4.0}+I{0.2}	K3P+I	1	0	16.045	20.755

31	DNA	0/0	K81{2.0,4.0}+G{0.5}	K3P+G4	1	2	18.938	18.966
32	DNA	0/0	K81{2.0,4.0}+I{0.2}+G{0.5}	K3P+I+G4	1	4	21.038	21.135
33	DNA	0/0	K81u{2.0,4.0}+F{0.3,0.1,0.2,0.4}	K3Pu+F+ASC	2	0	18.344	18.450
34	DNA	0/0	K81u{2.0,4.0}+F{0.2,0.1,0.3,0.4}+I{0.2}	K3Pu+F+I	1	6	20.120	21.044
35	DNA	0/0	K81u{2.0,4.0}+F{0.4,0.1,0.2,0.3}+G{0.5}	K3Pu+F+G4	1	0	18.970	19.023
36	DNA	0/0	K81u{2.0,4.0}+F{0.1,0.2,0.4,0.3}+I{0.2}+G{0.5}	K3Pu+F+I+G4	1	0	20.168	20.138
37	DNA	0/0	TPM2{2.0,4.0}	TPM2+ASC	2	0	19.333	19.401
38	DNA	0/0	TPM2{2.0,4.0}+I{0.2}	TPM2+I	1	0	19.165	20.671
39	DNA	0/0	TPM2{2.0,4.0}+G{0.5}	TPM2+G4	1	2	19.309	19.355
40	DNA	0/0	TPM2{2.0,4.0}+I{0.2}+G{0.5}	TPM2+I+G4	1	0	20.994	20.874
41	DNA	0/0	TPM2u{2.0,4.0}+F{0.3,0.1,0.2,0.4}	TPM2u+F+ASC	2	0	18.087	18.118
42	DNA	0/0	TPM2u{2.0,4.0}+F{0.2,0.1,0.3,0.4}+I{0.2}	TPM2u+F+I	1	2	19.714	21.628
43	DNA	0/0	TPM2u{2.0,4.0}+F{0.4,0.1,0.2,0.3}+G{0.5}	TPM2u+F+G4	1	0	19.905	19.956
44	DNA	0/0	TPM2u{2.0,4.0}+F{0.1,0.2,0.4,0.3}+I{0.2}+G{0.5}	TPM2u+F+I+G4	1	6	21.678	21.745
45	DNA	0/0	TPM3{2.0,4.0}	TPM3+ASC	2	0	19.080	19.057
46	DNA	0/0	TPM3{2.0,4.0}+I{0.2}	TPM3+I	1	4	18.682	20.628
47	DNA	0/0	TPM3{2.0,4.0}+G{0.5}	TPM3+G4	1	4	20.349	20.445

48	DNA	0/0	TPM3{2.0,4.0}+I{0.2}+G{0.5}	TPM3+I+G4	1	4	20.238	20.227
49	DNA	0/0	TPM3u{2.0,4.0}+F{0.3,0.1,0.2,0.4}	TPM3u+F+ASC	2	2	20.047	19.996
50	DNA	0/0	TPM3u{2.0,4.0}+F{0.2,0.1,0.3,0.4}+I{0.2}	TPM3u+F+I	1	0	21.102	19.425
51	DNA	0/0	TPM3u{2.0,4.0}+F{0.4,0.1,0.2,0.3}+G{0.5}	TPM3u+F+G4	1	2	18.297	18.307
52	DNA	0/0	TPM3u{2.0,4.0}+F{0.1,0.2,0.4,0.3}+I{0.2}+G{ 0.5}	TPM3u+F+I+G4	1	0	20.356	20.499
53	DNA	0/0	TIM{2.0,4.0,6.0}+F{0.3,0.1,0.2,0.4}	TIM+F+ASC	2	6	20.878	20.975
54	DNA	0/0	TIM{2.0,4.0,6.0}+F{0.2,0.1,0.3,0.4}+I{0.2}	TIM+F+I	1	10	17.254	18.367
55	DNA	0/0	TIM{2.0,4.0,6.0}+F{0.4,0.1,0.2,0.3}+G{0.5}	TIM+F+G4	1	0	19.885	19.890
56	DNA	0/0	TIM{2.0,4.0,6.0}+F{0.1,0.2,0.4,0.3}+I{0.2}+G{ 0.5}	TIM+F+I+G4	1	0	18.382	18.565
57	DNA	0/0	TIMe{2.0,4.0,6.0}	TIMe+ASC	2	0	19.395	19.344
58	DNA	0/0	TIMe{2.0,4.0,6.0}+I{0.2}	TIMe+I	1	4	19.829	18.809
59	DNA	0/0	TIMe{2.0,4.0,6.0}+G{0.5}	TIMe+G4	1	6	19.586	19.644
60	DNA	0/0	TIMe{2.0,4.0,6.0}+I{0.2}+G{0.5}	TIMe+I+G4	1	2	20.502	20.569
61	DNA	0/0	TIM2{2.0,4.0,6.0}+F{0.3,0.1,0.2,0.4}	TIM2+F+ASC	2	4	17.652	17.697
62	DNA	0/0	TIM2{2.0,4.0,6.0}+F{0.2,0.1,0.3,0.4}+I{0.2}	TIM2+F+I	1	6	20.355	21.957
63	DNA	0/0	TIM2{2.0,4.0,6.0}+F{0.4,0.1,0.2,0.3}+G{0.5}	TIM2+F+G4	1	0	18.273	18.273
64	DNA	0/0	TIM2{2.0,4.0,6.0}+F{0.1,0.2,0.4,0.3}+I{0.2}+G {0.5}	TIM2+F+I+G4	1	2	19.363	19.317

65	DNA	0/0	TIM2e{2.0,4.0,6.0}	TIM2e+ASC	2	8	18.393	18.371
66	DNA	0/0	TIM2e{2.0,4.0,6.0}+I{0.2}	TIM2e+I	1	2	15.628	19.874
67	DNA	0/0	TIM2e{2.0,4.0,6.0}+G{0.5}	TIM2e+G4	1	2	19.427	19.529
68	DNA	0/0	TIM2e{2.0,4.0,6.0}+I{0.2}+G{0.5}	TIM2e+I+G4	1	2	19.230	19.195
69	DNA	0/0	TIM3{2.0,4.0,6.0}+F{0.3,0.1,0.2,0.4}	TIM3+F+ASC	2	6	19.211	19.229
70	DNA	0/0	TIM3{2.0,4.0,6.0}+F{0.2,0.1,0.3,0.4}+I{0.2}	TIM3+F+I	1	4	21.319	20.223
71	DNA	0/0	TIM3{2.0,4.0,6.0}+F{0.4,0.1,0.2,0.3}+G{0.5}	TIM3+F+G4	1	4	19.340	19.439
72	DNA	0/0	TIM3{2.0,4.0,6.0}+F{0.1,0.2,0.4,0.3}+I{0.2}+G {0.5}	TIM3+F+I+G4	1	2	19.978	19.941
73	DNA	0/0	TIM3e{2.0,4.0,6.0}	TIM3e+ASC	2	4	18.704	18.744
74	DNA	0/0	TIM3e{2.0,4.0,6.0}+I{0.2}	TIM3e+I	1	2	17.797	19.202
75	DNA	0/0	TIM3e{2.0,4.0,6.0}+G{0.5}	TIM3e+G4	1	0	17.577	17.573
76	DNA	0/0	TIM3e{2.0,4.0,6.0}+I{0.2}+G{0.5}	TIM3e+I+G4	1	4	19.928	19.815
77	DNA	0/0	TVM{2.0,4.0,6.0,8.0}+F{0.3,0.1,0.2,0.4}	TVM+F+ASC	2	0	20.090	20.103
78	DNA	0/0	TVM{2.0,4.0,6.0,8.0}+F{0.2,0.1,0.3,0.4}+I{0.2 }	TVM+F+I	1	4	20.894	19.587
79	DNA	0/0	TVM{2.0,4.0,6.0,8.0}+F{0.4,0.1,0.2,0.3}+G{0. 5}	TVM+F+G4	1	0	19.045	19.168
80	DNA	0/0	TVM{2.0,4.0,6.0,8.0}+F{0.1,0.2,0.4,0.3}+I{0.2 }+G{0.5}	TVM+F+I+G4	1	2	19.861	19.905

81	DNA	0/0	TVMe{2.0,4.0,6.0,8.0}	TVMe+ASC	2	2	21.532	21.519
82	DNA	0/0	TVMe{2.0,4.0,6.0,8.0}+I{0.2}	TVMe+I	1	0	20.474	20.589
83	DNA	0/0	TVMe{2.0,4.0,6.0,8.0}+G{0.5}	TVMe+G4	1	0	18.789	18.976
84	DNA	0/0	TVMe{2.0,4.0,6.0,8.0}+I{0.2}+G{0.5}	TVMe+I+G4	1	6	19.383	19.419
85	DNA	0/0	SYM{2.0,4.0,6.0,8.0,10.0}	SYM+ASC	2	0	20.516	20.523
86	DNA	0/0	SYM{2.0,4.0,6.0,8.0,10.0}+I{0.2}	SYM+I	1	2	20.440	20.416
87	DNA	0/0	SYM{2.0,4.0,6.0,8.0,10.0}+G{0.5}	SYM+G4	1	4	19.600	19.808
88	DNA	0/0	SYM{2.0,4.0,6.0,8.0,10.0}+I{0.2}+G{0.5}	SYM+I+G4	1	4	19.429	19.317
89	Protein	0/0	Blosum62	Blosum62	1	6	19.568	19.507
90	Protein	0/0	cpREV	cpREV	1	4	16.273	16.165
91	Protein	0/0	Dayhoff	DCMut	2	4	22.340	22.375
92	Protein	0/0	DCMut	Dayhoff	2	2	21.328	21.333
93	Protein	0/0	FLU	FLU	1	2	17.545	17.511
94	Protein	0/0	HIVb	HIVb	1	0	20.710	20.578
95	Protein	0/0	HIVw	HIVw	1	6	20.923	20.782
96	Protein	0/0	JTT	JTT	1	4	18.371	18.533
97	Protein	0/0	JTTDCMut	JTTDCMut	1	12	20.420	20.741
98	Protein	0/0	LG	LG	1	2	22.356	22.245

99	Protein	0/0	mtART	mtART	1	4	17.961	18.250
100	Protein	0/0	mtMAM	mtMAM	1	2	18.460	18.556
101	Protein	0/0	mtREV	mtREV	1	8	18.682	18.737
102	Protein	0/0	mtZOA	mtZOA	1	12	19.678	19.724
103	Protein	0/0	mtMet	mtMet	1	4	23.644	23.674
104	Protein	0/0	mtVer	mtVer	1	4	17.670	17.727
105	Protein	0/0	mtInv	mtInv	1	6	19.786	19.849
106	Protein	0/0	Poisson	Poisson	1	2	18.748	18.896
107	Protein	0/0	PMB	PMB	1	4	21.342	21.231
108	Protein	0/0	rtREV	rtREV	1	2	18.487	18.679
109	Protein	0/0	VT	VT	1	2	18.501	18.369
110	Protein	0/0	WAG	WAG	1	2	20.125	20.276
111	Protein	0/0	GTR20+F	GTR20+F	1	2	21.327	21.343
112	Codon	0/0	MG{1.0}+F3X4{0.2,0.3,0.4,0.1,0.4,0.3,0.2,0.1,0.1,0.2,0.3,0.4}	MG+F3X4	1	0	18.759	18.822
113	Codon	0/0	MGK{1.0,0.3}+F3X4{0.2,0.3,0.4,0.1,0.4,0.3,0.2,0.1,0.1,0.2,0.3,0.4}	MG+F3X4	2	0	22.478	22.445
114	Codon	0/0	MG1KTS{1.0,0.3}+F3X4{0.2,0.3,0.4,0.1,0.4,0.3,0.2,0.1,0.1,0.2,0.3,0.4}	MG+F3X4	3	2	19.738	19.645

115	Codon	0/0	MG1KTV{1.0,0.3}+F3X4{0.2,0.3,0.4,0.1,0.4,0.3,0.2,0.1,0.1,0.2,0.3,0.4}	MG1KTV+F3X4	1	6	19.373	19.238
116	Codon	0/0	GY{1.0,0.3}+F	GY+F	1	2	19.894	19.809
117	Codon	0/0	GY1KTS{1.0,0.3}+F	GY+F	2	0	19.881	19.746
118	Codon	0/0	GY1KTV{1.0,0.3}+F	GY1KTV+F	1	0	20.767	20.937
119	Codon	0/0	GY2K{1.0,0.3,0.5}+F	GY+F	2	0	18.625	18.433
120	Codon	0/0	ECMK07+F3X4{0.2,0.3,0.4,0.1,0.4,0.3,0.2,0.1,0.1,0.2,0.3,0.4}+R3{0.5,2.0,0.2,3.5,0.3,0.5}	KOSI07+F3X4+R4	4	6	25.143	19.581
121	Codon	0/0	ECMrest+F3X4{0.2,0.3,0.4,0.1,0.4,0.3,0.2,0.1,0.1,0.2,0.3,0.4}+R3{0.5,2.0,0.2,3.5,0.3,0.5}	ECMrest+F3X4+R4	3	6	22.503	18.949
122	Codon	0/0	ECMS05+F3X4{0.2,0.3,0.4,0.1,0.4,0.3,0.2,0.1,0.1,0.2,0.3,0.4}+R3{0.5,2.0,0.2,3.5,0.3,0.5}	GY+F3X4+R5	8	4	22.606	20.269
123	Codon	0/0	ECMK07_GY2K{1.0,0.3,0.5}+F+R3{0.5,2.0,0.2,3.5,0.3,0.5}	ECMK07_GY2K+F+R5	13	2	22.528	22.401
124	Morphological	0/0	MK	MK+FQ	1	0	19.749	19.779
125	Morphological	0/0	MK+I{0.2}	MK+FQ+I	1	0	20.924	21.043
126	Morphological	0/0	MK+G{0.5}	MK+FQ+G4	1	0	18.694	18.666
127	Morphological	0/0	MK+I{0.2}+G{0.5}	MK+FQ+I+G4	1	0	19.503	19.407
128	Morphological	0/0	ORDERED	ORDERED+FQ+ASC	2	0	21.046	21.035

129	Morpho logical	0/0	ORDERED+I{0.2}	ORDERED+FQ +I	1	2	19.734	19.803
130	Morpho logical	0/0	ORDERED+G{0.5}	ORDERED+FQ +G4	1	0	20.130	20.120
131	Morpho logical	0/0	ORDERED+I{0.2}+G{0.5}	ORDERED+FQ +I+G4	1	2	18.243	18.053
132	Binary	0/0	JC2	JC2+FQ+ASC	2	8	22.133	22.067
133	Binary	0/0	JC2+I{0.2}	JC2+FQ+I	1	2	19.783	20.044
134	Binary	0/0	JC2+G{0.5}	JC2+FQ+G4	1	2	21.888	21.809
135	Binary	0/0	JC2+I{0.2}+G{0.5}	JC2+FQ+I+G4	1	2	20.121	20.073
136	Binary	0/0	GTR2+F{0.7/0.3}	GTR2+FO+ASC	2	4	22.119	22.103
137	Binary	0/0	GTR2+F{0.7/0.3}+I{0.2}	GTR2+FO+I	1	12	20.829	20.797
138	Binary	0/0	GTR2+F{0.7/0.3}+G{0.5}	GTR2+FO+G4	1	2	20.547	20.574
139	Binary	0/0	GTR2+F{0.7/0.3}+I{0.2}+G{0.5}	GTR2+FO+I+G 4	1	0	18.835	19.043

Lie Markov models

140	Protein	0/0	1.1	JC+ASC	8	2	19.677	19.797
141	Protein	0/0	RY10.12{0.5,0.6,0.9,0.2,0.1,0.4,0.7,0.8,0.3}+F{ 0.2/0.3/0.1/0.4}	RY10.12+ASC	2	0	18.151	18.826
142	Protein	0/0	WS10.12{-0.5,0.6,0.9,0.2,-0.1,0.4,0.7,-0.8,- 0.3}+F{0.2/0.3/0.1/0.4}	UNREST+FO+ ASC	6	2	18.773	19.003

143	Protein	0/0	MK10.12{0.5,0.6,0.9,0.2,0.1,0.4,0.7,0.8,0.3}+F{0.2/0.3/0.1/0.4}	UNREST+FO+ASC	4	2	17.920	18.524
144	Protein	0/0	RY10.34{0.5,-0.6,0.8,0.2,-0.1,0.4,0.7,-0.8,-0.35}+F{0.2/0.3/0.1/0.4}	UNREST+FO+ASC	4	2	17.901	18.240
145	Protein	0/0	WS10.34{-0.3,0.15,-0.9,0.2,0.6,-0.4,-0.7,0.8,0.5}+F{0.2/0.3/0.1/0.4}	UNREST+FO+ASC	6	0	17.694	21.320
146	Protein	0/0	MK10.34{0.8,-0.5,0.9,-0.35,-0.1,0.4,-0.75,0.6,-0.2}+F{0.2/0.3/0.1/0.4}	UNREST+FO+ASC	6	0	21.224	21.189
147	Protein	0/0	12.12{0.5,0.6,0.9,0.2,0.1,0.4,0.7,0.8,0.3,0.15,0.65}+F{0.2/0.3/0.1/0.4}	UNREST+FO+ASC	4	0	19.762	19.232
148	Protein	0/0	RY2.2b{0.2}	K2P+ASC	12	0	20.637	20.689
149	Protein	0/0	WS2.2b{0.6}	K3P+ASC	4	2	17.860	17.887
150	Protein	0/0	MK2.2b{0.6}	K3P+ASC	4	0	20.350	20.433
151	Protein	0/0	3.3a{0.6,-0.5}	K3P+ASC	6	0	19.513	19.647
152	Protein	0/0	RY3.3b{0.6,0.9}	RY3.3b+ASC	2	0	19.245	19.307
153	Protein	0/0	WS3.3b{0.6,0.9}	WS3.3b+ASC	2	0	20.406	20.401
154	Protein	0/0	MK3.3b{0.6,0.9}	MK3.3b+ASC	2	2	21.372	21.307
155	Protein	0/0	RY3.3c{-0.2,0.9}	TNe+ASC	10	2	18.676	18.710
156	Protein	0/0	WS3.3c{0.6,0.1}	WS3.3c+ASC	2	0	19.210	19.181
157	Protein	0/0	MK3.3c{0.6,0.9}	MK3.3c	1	0	21.364	21.378
158	Protein	0/0	RY3.4{-0.6,0.1}+F{0.3}	RY3.4+ASC	2	6	20.000	19.899

159	Protein	0/0	WS3.4{-0.6,0.9}+F{0.3}	WS3.4+ASC	2	2	19.134	19.446
160	Protein	0/0	MK3.4{0.6,-0.5}+F{0.3}	MK3.4+ASC	2	2	20.333	21.252
161	Protein	0/0	4.4a{0.6,-0.5,0.1}+F{0.2/0.3/0.1/0.4}	UNREST+FO+ ASC	4	2	15.938	18.585
162	Protein	0/0	RY4.4b{0.1,-0.5,0.7}+F{0.3}	RY4.4b+ASC	2	2	13.972	16.428
163	Protein	0/0	WS4.4b{0.1,-0.5,-0.1}+F{0.3}	WS4.4b+ASC	2	2	20.003	18.856
164	Protein	0/0	MK4.4b{-0.6,0.7,0.1}+F{0.3}	MK4.4b+ASC	2	2	20.403	20.674
165	Protein	0/0	RY4.5a{0.6,0.9,0.2}+F{0.3}	RY4.5a+ASC	2	0	19.763	19.721
166	Protein	0/0	WS4.5a{0.6,0.9,0.2}+F{0.3}	WS4.5a+ASC	2	0	21.153	21.085
167	Protein	0/0	MK4.5a{0.6,0.9,0.2}+F{0.3}	MK4.5a+ASC	2	4	20.536	20.532
168	Protein	0/0	RY4.5b{0.6,0.9,0.2}+F{0.3}	RY4.5b+ASC	2	2	20.246	20.290
169	Protein	0/0	WS4.5b{0.6,0.9,0.2}+F{0.3}	WS4.5b+ASC	2	2	21.117	21.115
170	Protein	0/0	MK4.5b{0.6,-0.9,0.2}+F{0.3}	MK4.5b+ASC	2	0	19.579	19.562
171	Protein	0/0	RY5.11a{0.5,-0.6,0.9,-0.25}+F{0.3/0.1}	UNREST+FO+ ASC	6	2	20.342	20.693
172	Protein	0/0	WS5.11a{0.5,0.6,0.9,0.2}+F{0.3/0.1}	UNREST+FO+ ASC	4	0	19.621	20.150
173	Protein	0/0	MK5.11a{0.5,0.6,0.9,0.2}+F{0.3/0.1}	UNREST+FO+ ASC	4	0	19.335	19.943
174	Protein	0/0	RY5.11b{0.5,0.6,0.9,0.2}	RY5.11b+ASC	2	0	20.044	20.066

175	Protein	0/0	WS5.11b{0.5,0.6,0.9,0.2}	WS5.11b+ASC	2	0	17.502	17.481
176	Protein	0/0	MK5.11b{0.5,0.6,0.9,0.2}	MK5.11b+ASC	2	2	23.573	23.650
177	Protein	0/0	RY5.11c{0.5,0.6,0.9,0.2}	RY5.11c+ASC	2	4	18.108	18.097
178	Protein	0/0	WS5.11c{0.5,0.6,0.9,0.2}	WS5.11c+ASC	2	0	17.608	17.614
179	Protein	0/0	MK5.11c{0.5,0.6,0.9,0.2}	MK5.11c+ASC	2	0	19.834	19.838
180	Protein	0/0	RY5.16{0.5,0.6,0.9,0.2}+F{0.3}	RY5.16+ASC	2	0	20.847	20.799
181	Protein	0/0	WS5.16{0.5,0.6,0.9,0.2}+F{0.3}	WS5.16+ASC	2	2	19.756	19.715
182	Protein	0/0	MK5.16{0.5,0.6,0.9,0.2}+F{0.3}	MK5.16+ASC	2	0	19.989	20.067
183	Protein	0/0	RY5.6a{0.5,0.6,0.9,0.2}	RY5.6a+ASC	2	0	21.334	21.199
184	Protein	0/0	WS5.6a{0.5,0.6,0.9,0.2}	WS5.6a+ASC	2	0	20.376	20.483
185	Protein	0/0	MK5.6a{0.55,-0.6,0.9,0.25}	MK5.6a+ASC	2	0	21.753	21.814
186	Protein	0/0	RY5.6b{0.5,-0.6,0.9,-0.2}+F{0.2/0.3/0.1/0.4}	RY5.6b+ASC	2	2	20.119	20.252
187	Protein	0/0	WS5.6b{0.15,-0.4,-0.8,0.5}+F{0.2/0.3/0.1/0.4}	UNREST+FO+ ASC	6	2	18.836	19.335
188	Protein	0/0	MK5.6b{-0.2,-0.65,0.9,-0.8}+F{0.2/0.3/0.1/0.4}	UNREST+FO+ ASC	6	2	18.799	19.695
189	Protein	0/0	RY5.7a{0.5,0.6,0.9,0.2}+F{0.3/0.1}	UNREST+FO+ ASC	4	4	17.356	17.636
190	Protein	0/0	WS5.7a{0.5,0.6,0.9,0.2}+F{0.3/0.1}	UNREST+FO+ ASC	4	0	18.799	19.024

191	Protein	0/0	MK5.7a{0.5,0.6,0.9,0.2}+F{0.3/0.1}	UNREST+FO+ ASC	4	6	17.740	17.957
192	Protein	0/0	RY5.7b{0.5,0.6,0.9,0.2}	RY5.7b+ASC	2	2	21.681	21.684
193	Protein	0/0	WS5.7b{0.5,0.6,0.9,0.2}	WS5.7b+ASC	2	4	22.597	22.567
194	Protein	0/0	MK5.7b{0.5,0.6,0.9,0.2}	MK5.7b+ASC	2	0	19.275	19.289
195	Protein	0/0	RY5.7c{0.5,0.6,0.9,0.2}	RY5.7c+ASC	2	2	23.344	23.285
196	Protein	0/0	WS5.7c{0.5,0.6,0.9,0.2}	WS5.7c+ASC	2	2	20.398	20.446
197	Protein	0/0	MK5.7c{0.5,0.6,0.9,0.2}	MK5.7c+ASC	2	2	19.571	19.605
198	Protein	0/0	RY6.17a{0.5,0.6,0.9,0.2,0.1}+F{0.3}	RY6.17a+ASC	2	2	19.157	19.206
199	Protein	0/0	WS6.17a{0.5,-0.6,-0.7,0.2,-0.1}+F{0.3}	WS6.17a+ASC	2	0	21.618	21.650
200	Protein	0/0	MK6.17a{0.5,0.6,0.9,0.2,0.1}+F{0.3}	MK6.17a+ASC	2	2	23.593	23.640
201	Protein	0/0	RY6.17b{0.5,0.6,0.9,0.2,0.1}+F{0.3}	RY6.17b+ASC	2	4	17.987	17.911
202	Protein	0/0	WS6.17b{0.5,0.6,0.9,0.2,0.1}+F{0.3}	WS6.17b+ASC	2	4	23.100	23.024
203	Protein	0/0	MK6.17b{0.5,0.6,0.9,0.2,0.1}+F{0.3}	MK6.17b+ASC	2	0	21.355	21.281
204	Protein	0/0	RY6.6{0.5,0.6,0.9,0.2,0.1}+F{0.3}	RY6.6+ASC	2	2	18.793	18.712
205	Protein	0/0	WS6.6{0.5,0.6,0.9,0.2,0.1}+F{0.3}	WS6.6+ASC	2	0	18.756	18.794
206	Protein	0/0	MK6.6{0.5,0.7,0.9,0.3,0.1}+F{0.3}	MK6.6+ASC	2	4	21.716	21.806
207	Protein	0/0	6.7a{0.5,-0.65,0.9,-0.25,0.1}+F{0.2/0.3/0.1/0.4}	UNREST+FO+ ASC	4	2	19.566	21.307

208	Protein	0/0	RY6.7b{-0.5,0.6,-0.9,0.2,-0.1}+F{0.2/0.3/0.1/0.4}	RY6.7b+ASC	2	2	16.927	16.619
209	Protein	0/0	WS6.7b{0.5,0.6,0.9,0.2,0.1}+F{0.2/0.3/0.1/0.4}	UNREST+FO+ASC	4	0	19.180	19.723
210	Protein	0/0	MK6.7b{0.55,-0.4,0.9,-0.2,0.1}+F{0.2/0.3/0.1/0.4}	UNREST+FO+ASC	6	0	16.825	17.920
211	Protein	0/0	RY6.8a{0.5,-0.6,0.7,0.2,-0.15}+F{0.2/0.3/0.1/0.4}	RY6.8a+ASC	2	2	15.825	18.221
212	Protein	0/0	WS6.8a{0.5,0.6,0.9,0.2,0.1}+F{0.2/0.3/0.1/0.4}	WS6.8a+ASC	2	8	17.771	18.188
213	Protein	0/0	MK6.8a{0.5,0.6,0.9,0.2,0.1}+F{0.2/0.3/0.1/0.4}	UNREST+FO+ASC	4	0	19.855	20.618
214	Protein	0/0	RY6.8b{0.5,0.6,0.9,0.2,0.1}+F{0.3}	RY6.8b+ASC	2	0	17.673	17.850
215	Protein	0/0	WS6.8b{0.5,0.6,0.9,0.2,0.1}+F{0.3}	WS6.8b+ASC	2	0	19.355	19.574
216	Protein	0/0	MK6.8b{0.5,0.6,0.9,0.2,0.1}+F{0.3}	MK6.8b+ASC	2	0	18.698	18.988
217	Protein	0/0	RY8.10a{0.5,-0.6,-0.9,-0.2,0.1,0.4,0.7}+F{0.2/0.3/0.1/0.4}	RY8.10a+ASC	2	2	16.952	17.422
218	Protein	0/0	WS8.10a{0.5,0.6,0.9,0.2,0.1,0.4,0.7}+F{0.2/0.3/0.1/0.4}	UNREST+FO+ASC	4	2	19.493	19.560
219	Protein	0/0	MK8.10a{0.5,0.6,0.9,0.2,0.1,0.4,0.7}+F{0.2/0.3/0.1/0.4}	UNREST+FO+ASC	4	4	20.861	21.537
220	Protein	0/0	RY8.10b{0.5,0.6,0.9,0.2,0.1,0.4,0.7}+F{0.3}	RY8.10b+ASC	2	4	20.006	20.449
221	Protein	0/0	WS8.10b{0.5,0.6,0.9,0.2,0.1,0.4,0.7}+F{0.3}	WS8.10b+ASC	2	0	18.852	19.275
222	Protein	0/0	MK8.10b{0.5,0.6,0.9,0.2,0.1,0.4,0.7}+F{0.3}	MK8.10b+ASC	2	0	19.882	20.367

223	Protein	0/0	RY8.16{0.5,0.6,0.9,0.2,0.1,0.4,0.7}+F{0.2/0.3/0.1/0.4}	UNREST+FO+ASC	4	4	21.246	20.620
224	Protein	0/0	WS8.16{0.5,0.6,0.9,0.2,0.1,0.4,0.7}+F{0.2/0.3/0.1/0.4}	WS8.16+ASC	2	0	18.289	19.226
225	Protein	0/0	MK8.16{-0.55,0.6,0.8,-0.2,0.1,0.4,-0.7}+F{0.2/0.3/0.1/0.4}	MK8.16+ASC	2	4	20.197	20.346
226	Protein	0/0	RY8.17{0.2,-0.6,0.9,0.5,-0.1,0.4,0.7}+F{0.2/0.3/0.1/0.4}	UNREST+FO+ASC	6	4	17.220	17.000
227	Protein	0/0	WS8.17{0.5,0.6,0.9,0.2,0.1,0.4,0.7}+F{0.2/0.3/0.1/0.4}	WS8.17+ASC	2	2	18.646	19.195
228	Protein	0/0	MK8.17{0.3,0.6,-0.9,0.2,-0.4,0.4,0.7}+F{0.2/0.3/0.1/0.4}	UNREST+FO+ASC	8	2	19.098	19.620
229	Protein	0/0	RY8.18{0.4,-0.6,0.9,-0.2,0.1,-0.5,0.7}+F{0.2/0.3/0.1/0.4}	UNREST+FO+ASC	4	0	20.931	21.249
230	Protein	0/0	WS8.18{0.5,0.6,0.9,0.2,0.1,0.4,0.7}+F{0.2/0.3/0.1/0.4}	WS8.18+ASC	2	2	21.260	22.280
231	Protein	0/0	MK8.18{0.5,0.85,-0.9,0.2,-0.15,0.4,-0.7}+F{0.2/0.3/0.1/0.4}	MK8.18+ASC	2	0	19.306	18.755
232	Protein	0/0	RY8.8{0.5,0.6,0.9,0.2,0.1,0.4,0.7}+F{0.2/0.3/0.1/0.4}	RY8.8+ASC	2	2	21.408	20.938
233	Protein	0/0	WS8.8{-0.5,0.6,-0.9,0.2,-0.1,0.4,-0.7}+F{0.2/0.3/0.1/0.4}	UNREST+FO+ASC	4	0	18.500	18.814
234	Protein	0/0	MK8.8{0.45,-0.6,0.8,0.3,-0.1,-0.3,-0.7}+F{0.2/0.3/0.1/0.4}	MK8.8+ASC	2	2	21.407	20.801
235	Protein	0/0	RY9.20a{0.9,-0.65,0.9,0.2,-0.1,0.4,0.7,-0.8}+F{0.3/0.1}	RY9.20a+ASC	2	2	20.044	19.519

236	Protein	0/0	WS9.20a{0.35,0.6,-0.9,0.2,0.1,-0.4,0.7,0.8}+F{0.3/0.1}	UNREST+FO+ASC	6	0	16.002	16.896
237	Protein	0/0	MK9.20a{0.5,0.6,0.9,0.2,0.1,0.4,0.7,0.8}+F{0.3/0.1}	MK9.20a+ASC	2	0	19.639	19.915
238	Protein	0/0	9.20b{0.5,0.6,0.9,0.2,0.1,0.4,0.7,0.8}	9.20b+ASC	2	4	20.319	20.329
**Mixture models**								
239	Protein	0/0	C10	C10	1	0	19.164	19.055
240	Protein	0/0	EX2	GTR20+F+R2	2	2	22.741	22.874
241	Protein	0/0	EX3	EX3+R2	5	0	22.118	21.008
242	Protein	0/0	EHO	EHO	1	0	18.576	18.627
243	Protein	0/0	UL2	UL2+R2	10	0	25.265	20.933
244	Protein	0/0	UL3	UL3+R2	5	0	22.850	20.000
245	Protein	0/0	EX_EHO	EX_EHO+R2	9	0	22.013	19.726
246	Protein	0/0	LG4M	LG4M	1	4	17.510	17.523
247	Protein	0/0	CF4	CF4+F+G	4	0	20.496	20.772
248	Protein	0/0	JTT+CF4+G4	JTT+CF4+F+G	3	0	20.777	20.209
249	DNA	0/0	MIX{JC,HKY{2.0}}+G4	MIX{JC,HKY+F}+G4	1	2	17.891	17.863

**Heterotachy models**								
250	DNA	0/0	GTR{2/3/4/5/6}+F{0.2/0.3/0.1/0.4}+H4{0.15/0.2/0.35/0.3}	GTR+FU+H4	1	0	21.524	21.599
251	DNA	0/0	MIX{GTR{2/3/4/5/6},GTR{6/2/5/4/7},GTR{4/7/6/2/8},GTR{5/2/7/6/9}}+F{0.2/0.3/0.1/0.4}*H4{0.15/0.2/0.35/0.3}	GTR*H4	1	0	26.843	26.872
252	DNA	0/0	MIX{GTR{2/3/4/5/6}+F{0.2/0.3/0.4/0.1},GTR{6/2/5/4/7}+F{0.3/0.2/0.1/0.4},GTR{4/7/6/2/8}+F{0.4/0.1/0.3/0.2},GTR{5/2/7/6/9}+F{0.1/0.2/0.4/0.3}}*H4{0.15/0.2/0.35/0.3}	GTR+FO*H4	1	0	20.897	20.902
**Insertion-deletion models**								
253	DNA	0/0.02	GTR{1.658853/3.559765/0.056937/1.192477/0.329238}+F{0.386134/0.22998/0.174036/0.20985}	GTR+F	1	0	21.453	21.385
254	DNA	0/0.04	GTR{1.32776/8.778905/0.84919/0.515804/1.901472}+F{0.172235/0.264059/0.264182/0.299524}	GTR+F	1	2	19.834	19.890
255	DNA	0/0.06	GTR{1.119447/3.559765/0.221697/1.014989/20.711653}+F{0.305724/0.243384/0.196665/0.254226}	GTR+F	1	0	19.936	19.769
256	DNA	0/0.08	GTR{0.086494/2.323423/2.809304/1.318319/4.171516}+F{0.284127/0.219497/0.186409/0.309967}	GTR+F	1	2	20.261	20.194
257	DNA	0/0.1	GTR{2.685037/24.868964/1.567923/0.101501/3.578998}+F{0.266817/0.202613/0.308702/0.221868}	GTR+F	1	2	19.554	19.706

258	DNA	0.02/0	GTR{8.193704/8.778905/3.613696/0.163775/31.523798}+F{0.292788/0.20068/0.175349/0.331184}	GTR+F	1	2	21.359	21.315
259	DNA	0.02/0.02	GTR{0.202229/4.955478/1.841897/1.864075/0.222629}+F{0.303056/0.194328/0.159597/0.343019}	GTR+F	1	4	21.550	21.543
260	DNA	0.02/0.04	GTR{8.193704/2.035624/0.340067/1.636181/214.150146}+F{0.273051/0.240699/0.189678/0.296573}	GTR+F	1	4	18.903	18.706
261	DNA	0.02/0.06	GTR{1.225831/0.318379/1.382544/5.206825/4.826486}+F{0.329796/0.339286/0.131282/0.199636}	GTR+F	1	0	20.049	20.150
262	DNA	0.02/0.08	GTR{2.666457/4.112417/0.177203/0.910549/10.179543}+F{0.243052/0.364955/0.187378/0.204614}	GTR+F	1	0	19.370	19.341
263	DNA	0.02/0.1	GTR{8.193704/7.74051/1.841897/0.841534/1.788653}+F{0.28858/0.320342/0.182192/0.208886}	GTR+F	1	2	19.725	19.811
264	DNA	0.04/0	GTR{1.259178/14.491652/0.84919/1.192477/17.608509}+F{0.364391/0.166043/0.209958/0.259609}	GTR+F	1	0	18.901	18.877
265	DNA	0.04/0.02	GTR{1.622091/3.548607/2.64403/0.902628/3.029666}+F{0.293661/0.223556/0.163246/0.319537}	GTR+F	1	2	21.292	21.255
266	DNA	0.04/0.04	GTR{1.841625/5.32596/2.941686/0.515804/5.660189}+F{0.134343/0.270785/0.293569/0.301302}	GTR+F	1	2	17.375	17.401

267	DNA	0.04/0.06	GTR{1.119447/7.159051/2.006633/0.975949/0.970356}+F{0.341057/0.27335/0.177055/0.208539}	GTR+F	1	6	20.031	19.986
268	DNA	0.04/0.08	GTR{2.379396/4.955478/0.989033/13.917175/10.179543}+F{0.216181/0.363212/0.16473/0.255877}	GTR+F	1	6	20.594	20.546
269	DNA	0.04/0.1	GTR{1.487735/1.53548/1.741463/1.695331/11.237555}+F{0.335028/0.192155/0.194448/0.278368}	GTR+F	1	4	22.080	22.037
270	DNA	0.06/0	GTR{1.492042/1.529111/0.876352/0.781435/9.382395}+F{0.320038/0.20406/0.201034/0.274867}	GTR+F	1	2	21.773	21.586
271	DNA	0.06/0.02	GTR{2.666457/4.955478/0.221697/1.521687/8.655391}+F{0.314747/0.227549/0.20465/0.253054}	GTR+F	1	4	17.989	18.032
272	DNA	0.06/0.04	GTR{1.119447/2.949316/0.478944/0.517493/17.483976}+F{0.357602/0.191959/0.165626/0.284813}	GTR+F	1	0	21.267	21.021
273	DNA	0.06/0.06	GTR{2.857435/9.196923/0.351809/4.999912/1.653673}+F{0.303202/0.195056/0.184061/0.317681}	GTR+F	1	0	20.698	20.676
274	DNA	0.06/0.08	GTR{2.857435/7.236792/0.525951/0.783632/5.026411}+F{0.23663/0.161377/0.376/0.225992}	GTR+F	1	2	20.575	20.645
275	DNA	0.06/0.1	GTR{0.346774/4.384402/2.905711/3.191284/8.655391}+F{0.329501/0.271937/0.21811/0.180452}	GTR+F	1	6	20.051	20.025
276	DNA	0.08/0	GTR{0.918148/7.28152/0.112665/0.975949/8.452647}+F{0.263484/0.318991/0.229091/0.188434}	GTR+F	1	2	21.958	21.939

277	DNA	0.08/0.02	GTR{3.806577/8.494684/1.094147/3.506839/3.227558}+F{0.38109/0.174324/0.22796/0.216625}	GTR+F	1	0	20.549	20.511
278	DNA	0.08/0.04	GTR{1.436101/5.051975/0.112665/0.930071/7.797079}+F{0.269989/0.245349/0.261474/0.223188}	GTR+F	1	0	19.126	19.082
279	DNA	0.08/0.06	GTR{0.389424/2.591557/0.546466/1.288688/20.711653}+F{0.340814/0.169713/0.160952/0.328521}	GTR+F	1	0	18.733	18.843
280	DNA	0.08/0.08	GTR{3.166275/24.868964/0.221697/0.470818/3.128172}+F{0.179181/0.20477/0.238503/0.377545}	GTR+F	1	2	21.869	21.728
281	DNA	0.08/0.1	GTR{5.08016/4.600208/1.346329/13.917175/7.797079}+F{0.291928/0.233534/0.139101/0.335437}	GTR+F	1	2	20.441	20.325
282	DNA	0.1/0	GTR{1.841625/7.236792/0.417861/1.099721/3.27437}+F{0.311121/0.268752/0.256148/0.163979}	GTR+F	1	0	20.703	20.755
283	DNA	0.1/0.02	GTR{5.08016/8.803904/2.809304/2.939371/2.492354}+F{0.298449/0.155919/0.216434/0.329199}	GTR+F	1	0	18.965	18.966
284	DNA	0.1/0.04	GTR{2.857435/3.344601/0.876352/2.455665/70.026968}+F{0.253814/0.204526/0.240511/0.301149}	GTR+F	1	6	21.104	21.135
285	DNA	0.1/0.06	GTR{5.08016/1.692792/0.431923/2.769857/8.655391}+F{0.379775/0.204224/0.160545/0.255456}	GTR+F	1	4	18.462	18.450

286	DNA	0.1/0.08	GTR{1.32776/7.185683/0.567838/13.917175/94.907071}+F{0.273149/0.350457/0.137704/0.238689}	GTR+F	1	4	20.904	21.044
287	DNA	0.1/0.1	GTR{1.644819/3.548607/1.174211/0.464509/3.519925}+F{0.220834/0.208764/0.324964/0.245438}	GTR+F	1	2	19.052	19.023

NOTE.-We simulated 287 MSAs from random 100-tip trees with sequence length of 10K sites (for DNA, binary, morphological, codon data) and 1K sites (for protein data) across a wide range of substitution models and insertion-deletion rates of 0.0, 0.02, 0.04, 0.06, 0.08 and 0.1. These choices of indel rates follow empirical studies (Cartwright 2009). We then ran IQ-TREE to determine the best-fit model using ModelFinder (Kalyaanamoorthy et al. 2017) and reconstructed phylogenetic trees under the best-fit model. We compared the topology between the true trees and the inferred trees using the Robinson-Foulds distance (Robinson and Foulds 1981). The table shows detailed results, each row corresponds to one test case. In 148 tests (51.57%), the true model was recovered as the best-fit model. In 243 tests (84.67%), 246 tests (85.71%), and 267 tests (93.03%), the true models appear in the top-2, top-3, and top-4 best models, respectively. The average Robinson-Foulds distance between the true trees and the inferred trees across all test cases was 2.06 (s.e. 0.135). That means the inferred trees differed from the true trees by only 1.03 out of 97 (1.06%) internal branches. The tree lengths (sum of branch lengths) of the inferred trees differed from the true trees by only 1.9%.

Table S2. Comparison results between AliSim and INDELible on simulating 35 MSAs with different combinations of insertion and deletion rates of 0.0, 0.02, 0.04, 0.06, 0.08 and 0.1.

**Indel-rate**	**The true (input) model**	**Alignment length**			**Proportion of gaps (%)**		
		**AliSim**	**INDELible**	**Difference**	**AliSim**	**INDELible**	**Difference**
0/0.02	GTR{1.658853/3.559765/0.056937/1.192477/0.329238}+F{0.386134/0.22998/0.174036/0.20985}	10000	10000	0.00%	2.85%	2.74%	0.11%
0/0.04	GTR{1.32776/8.778905/0.84919/0.515804/1.901472}+F{0.172235/0.264059/0.264182/0.299524}	10000	10000	0.00%	5.46%	5.88%	0.42%
0/0.06	GTR{1.119447/3.559765/0.221697/1.014989/20.711653}+F{0.305724/0.243384/0.196665/0.254226}	10000	10000	0.00%	7.33%	7.36%	0.03%

0/0.08	GTR{0.086494/2.323423/2.809304/1.318319/4.171516}+F{0.284127/0.219497/0.186409/0.309967}	10000	10000	0.00%	13.59%	13.55%	0.04%
0/0.1	GTR{2.685037/24.868964/1.567923/0.101501/3.578998}+F{0.266817/0.202613/0.308702/0.221868}	10000	10000	0.00%	10.59%	10.84%	0.25%
0.02/0	GTR{8.193704/8.778905/3.613696/0.163775/31.523798}+F{0.292788/0.20068/0.175349/0.331184}	15575	15593	0.12%	32.74%	32.95%	0.21%
0.02/0.02	GTR{0.202229/4.955478/1.841897/1.864075/0.222629}+F{0.303056/0.194328/0.159597/0.343019}	15347	15272	0.49%	34.95%	34.53%	0.42%
0.02/0.04	GTR{8.193704/2.035624/0.340067/1.636181/214.150146}+F{0.273051/0.240699/0.189678/0.296573}	14322	14459	0.95%	33.00%	33.36%	0.36%
0.02/0.06	GTR{1.225831/0.318379/1.382544/5.206825/4.826486}+F{0.329796/0.339286/0.131282/0.199636}	14760	14688	0.49%	37.01%	36.40%	0.61%
0.02/0.08	GTR{2.666457/4.112417/0.177203/0.910549/10.179543}+F{0.243052/0.364955/0.187378/0.204614}	14535	14416	0.83%	35.59%	34.89%	0.70%
0.02/0.1	GTR{8.193704/7.74051/1.841897/0.841534/1.788653}+F{0.28858/0.320342/0.182192/0.208886}	14465	14384	0.56%	38.50%	37.94%	0.56%
0.04/0	GTR{1.259178/14.491652/0.84919/1.192477/17.608509}+F{0.364391/0.166043/0.209958/0.259609}	19575	19777	1.02%	47.33%	47.88%	0.55%
0.04/0.02	GTR{1.622091/3.548607/2.64403/0.902628/3.029666}+F{0.293661/0.223556/0.163246/0.319537}	21088	20891	0.94%	50.78%	50.44%	0.34%
0.04/0.04	GTR{1.841625/5.32596/2.941686/0.515804/5.660189}+F{0.134343/0.270785/0.293569/0.301302}	18653	18607	0.25%	46.38%	46.28%	0.10%
0.04/0.06	GTR{1.119447/7.159051/2.006633/0.975949/0.970356}+F{0.341057/0.27335/0.177055/0.208539}	19937	19830	0.54%	50.79%	50.64%	0.15%
0.04/0.08	GTR{2.379396/4.955478/0.989033/13.917175/10.179543}+F{0.216181/0.363212/0.16473/0.255877}	19725	19793	0.34%	51.89%	51.95%	0.06%
0.04/0.1	GTR{1.487735/1.53548/1.741463/1.695331/11.237555}+F{0.335028/0.192155/0.194448/0.278368}	20136	20154	0.09%	54.53%	54.35%	0.18%
0.06/0	GTR{1.492042/1.529111/0.876352/0.781435/9.382395}+F{0.320038/0.20406/0.201034/0.274867}	27383	27412	0.11%	60.42%	60.46%	0.04%
0.06/0.02	GTR{2.666457/4.955478/0.221697/1.521687/8.655391}+F{0.314747/0.227549/0.20465/0.253054}	24312	24790	1.93%	55.59%	56.38%	0.79%

0.06/0.04	GTR{1.119447/2.949316/0.478944/0.517493/17.483976}+F{0.357602/0.191959/0.165626/0.284813}	26281	26085	0.75%	60.84%	60.60%	0.24%
0.06/0.06	GTR{2.857435/9.196923/0.351809/4.999912/1.653673}+F{0.303202/0.195056/0.184061/0.317681}	25504	25545	0.16%	60.79%	60.83%	0.04%
0.06/0.08	GTR{2.857435/7.236792/0.525951/0.783632/5.026411}+F{0.23663/0.161377/0.376/0.225992}	25008	25288	1.11%	61.00%	61.50%	0.50%
0.06/0.1	GTR{0.346774/4.384402/2.905711/3.191284/8.655391}+F{0.329501/0.271937/0.21811/0.180452}	24276	24320	0.18%	60.74%	60.79%	0.05%
0.08/0	GTR{0.918148/7.28152/0.112665/0.975949/8.452647}+F{0.263484/0.318991/0.229091/0.188434}	34673	34407	0.77%	67.35%	67.41%	0.06%
0.08/0.02	GTR{3.806577/8.494684/1.094147/3.506839/3.227558}+F{0.38109/0.174324/0.22796/0.216625}	32115	31698	1.32%	66.30%	65.85%	0.45%
0.08/0.04	GTR{1.436101/5.051975/0.112665/0.930071/7.797079}+F{0.269989/0.245349/0.261474/0.223188}	29701	29675	0.09%	64.99%	65.11%	0.12%
0.08/0.06	GTR{0.389424/2.591557/0.546466/1.288688/20.711653}+F{0.340814/0.169713/0.160952/0.328521}	29468	29016	1.56%	65.08%	64.65%	0.43%
0.08/0.08	GTR{3.166275/24.868964/0.221697/0.470818/3.128172}+F{0.179181/0.20477/0.238503/0.377545}	31284	31654	1.17%	68.21%	68.46%	0.25%
0.08/0.1	GTR{3.049122/8.778905/0.654595/0.586987/0.329238}+F{0.380216/0.183774/0.164769/0.271242}	29772	29899	0.42%	67.18%	67.26%	0.08%
0.1/0	GTR{1.841625/7.236792/0.417861/1.099721/3.27437}+F{0.311121/0.268752/0.256148/0.163979}	38743	38627	0.30%	71.25%	71.16%	0.09%
0.1/0.02	GTR{5.08016/8.803904/2.809304/2.939371/2.492354}+F{0.298449/0.155919/0.216434/0.329199}	37179	36910	0.73%	68.81%	68.76%	0.05%
0.1/0.04	GTR{2.857435/3.344601/0.876352/2.455665/70.026968}+F{0.253814/0.204526/0.240511/0.301149}	39109	39100	0.02%	71.50%	71.48%	0.02%
0.1/0.06	GTR{5.08016/1.692792/0.431923/2.769857/8.655391}+F{0.379775/0.204224/0.160545/0.255456}	34159	34184	0.07%	69.20%	69.12%	0.08%
0.1/0.08	GTR{1.32776/7.185683/0.567838/13.917175/94.907071}+F{0.273149/0.350457/0.137704/0.238689}	36806	36680	0.34%	72.29%	72.24%	0.05%
0.1/0.1	GTR{0.975527/7.153133/0.59506/11.3205/10.321999}+F{0.240426/0.200113/0.322616/0.236845}	33561	33751	0.56%	70.32%	70.53%	0.21%

NOTE.- We simulated 10K-site MSAs from 100-tip trees under the general time-reversible (GTR) model (Tavaré 1986) and used the negative binomial distribution (with a mean of 1.25) to generate the insertion and deletion lengths. Each row shows the alignment length and the proportion of gaps of the simulated MSA for each indel-rate combination. The average differences in the alignment length and proportion of gaps between MSAs simulated by AliSim and those by INDELible were 0.52% and 0.25%, respectively.

Table S3. Switching parameters for different sequence lengths, ranging from 1K to 100K sites with/without continuous rate heterogeneity.

Model	#Site	Runtime of the Probability matrix approach (ms)	Range of branch length	Switching params
GTR+F	1K	342.841	0.0001-0.1	0.002215
GTR+F	2K	605.015	0.0001-0.1	0.001097
GTR+F	3K	872.343	0.0001-0.1	0.000754
GTR+F	4K	1159.497	0.0001-0.1	0.000579
GTR+F	5K	1425.972	0.0001-0.1	0.000451
GTR+F	6K	1687.084	0.0001-0.1	0.000384
GTR+F	7K	1985.005	0.0001-0.1	0.000335
GTR+F	8K	2271.611	0.0001-0.1	0.000294
GTR+F	9K	2551.453	0.0001-0.1	0.000262
GTR+F	10K	2811.463	1e-05-0.01	0.000232
GTR+F	20K	5666.547	1e-05-0.01	0.000116
GTR+F	30K	8574.731	1e-05-0.01	0.000074
GTR+F	40K	11338.240	1e-05-0.01	0.000052
GTR+F	50K	14101.600	1e-05-0.01	0.000035
GTR+F	60K	17025.880	1e-05-0.01	0.000027
GTR+F	70K	19920.400	1e-05-0.01	0.000026
GTR+F	80K	22567.150	1e-05-0.01	0.000021
GTR+F	90K	25515.960	1e-05-0.01	0.000016
GTR+F	100K	28254.480	1e-06-0.001	0.000014
GTR+F+GC	1K	1433.839	0.0001-0.1	0.01327
GTR+F+GC	2K	2788.569	0.0001-0.1	0.006695
GTR+F+GC	3K	4153.132	0.0001-0.1	0.004471
GTR+F+GC	4K	5506.709	0.0001-0.1	0.00334
GTR+F+GC	5K	6859.667	0.0001-0.1	0.002671
GTR+F+GC	6K	8213.643	0.0001-0.1	0.002245
GTR+F+GC	7K	9573.880	0.0001-0.1	0.001908
GTR+F+GC	8K	10943.020	0.0001-0.1	0.001675
GTR+F+GC	9K	12308.660	0.0001-0.1	0.001472
GTR+F+GC	10K	13669.790	1e-05-0.01	0.001328

GTR+F+GC	20K	27292.360	1e-05-0.01	0.000653
GTR+F+GC	30K	40929.470	1e-05-0.01	0.000433
GTR+F+GC	40K	54561.300	1e-05-0.01	0.000301
GTR+F+GC	50K	68394.600	1e-05-0.01	0.0002
GTR+F+GC	60K	81908.780	1e-05-0.01	0.000164
GTR+F+GC	70K	95600.800	1e-05-0.01	0.000144
GTR+F+GC	80K	109123.800	1e-05-0.01	0.00012
GTR+F+GC	90K	122608.600	1e-05-0.01	0.000105
GTR+F+GC	100K	136422.700	1e-05-0.01	0.000091

NOTE.- Each row shows the model, the sequence length, the runtime of the probability matrix approach, the range of branch length to apply the binary search, and the switching parameter.

References

Cartwright RA. 2009. Problems and solutions for estimating indel rates and length distributions. *Mol Biol Evol*. 26(2):473–480.

De Maio Nicola, Weilguny L, Walker CR, Turakhia Y, Corbett-Detig R, Goldman N. 2021. phastSim: efficient simulation of sequence evolution for pandemic-scale datasets. *bioRxiv*. doi: 10.1101/2021.03.15.435416.

Fletcher W, Yang Z. 2009. INDELible: A flexible simulator of biological sequence evolution. *Mol Biol Evol*. 26(8):1879–1888.

Goldman N, Yang Z. 1994. A codon-based model of nucleotide substitution for protein-coding DNA sequences. *Mol Biol Evol.* 11(5):725–736.

Kalyaanamoorthy S, Minh BQ, Wong TKF, von Haeseler A, Jermiin LS. 2017. ModelFinder: Fast model selection for accurate phylogenetic estimates. *Nat Methods*. 14(6):587–589.

Robinson DF, Foulds LR. 1981. Comparison of phylogenetic trees. *Math Biosci*. 53(1–2):131–147.

Tavaré S. Miura RM 1986. Some probabilistic and statistical problems in the analysis of DNA sequences. *Lect Math life Sci*. 17:57–86.
